# Vicarious Radiometric Calibration of Ocean Color Bands for FY-3D/MERSI-II at Lake Qinghai, China

**DOI:** 10.3390/s21010139

**Published:** 2020-12-28

**Authors:** Shengli Chen, Xiaobing Zheng, Xin Li, Wei Wei, Shenda Du, Fuxiang Guo

**Affiliations:** 1 Key Laboratory of Optical Calibration and Characterization, Anhui Institute of Optics and Fine Mechanics, Hefei Institutes of Physical Science, Chinese Academy of Sciences, Hefei 230031, China; chenshl@mail.ustc.edu.cn (S.C.); xli@aiofm.ac.cn (X.L.); weiwei@aiofm.ac.cn (W.W.); dushenda@mail.ustc.edu.cn (S.D.); gfx@mail.ustc.edu.cn (F.G.); 2 University of Science and Technology of China, Hefei 230026, China

**Keywords:** vicarious calibration, ocean color, water-leaving radiance, radiative transfer

## Abstract

To calibrate the low signal response of the ocean color (OC) bands and test the stability of the Fengyun-3D (FY-3D)/Medium Resolution Spectral Imager II (MERSI-II), an absolute radiometric calibration field test of FY-3D/MERSI-II at the Lake Qinghai Radiometric Calibration Site (RCS) was carried out in August 2018. The lake surface and atmospheric parameters were mainly measured by advanced observation instruments, and the MODerate spectral resolution atmospheric TRANsmittance algorithm and computer model (MODTRAN4.0) was used to simulate the multiple scattering radiance value at the altitude of the sensor. The results showed that the relative deviations between bands 9 and 12 are within 5.0%, while the relative deviations of bands 8, and 13 are 17.1%, and 12.0%, respectively. The precision of the calibration method was verified by calibrating the Aqua/Moderate-resolution Imaging Spectroradiometer (MODIS) and National Polar-orbiting Partnership (NPP)/Visible Infrared Imaging Radiometer (VIIRS), and the deviation of the calibration results was evaluated with the results of the Dunhuang RCS calibration and lunar calibration. The results showed that the relative deviations of NPP/VIIRS were within 7.0%, and the relative deviations of Aqua/MODIS were within 4.1% from 400 nm to 600 nm. The comparisons of three on-orbit calibration methods indicated that band 8 exhibited a large attenuation after launch and the calibration results had good consistency at the other bands except for band 13. The uncertainty value of the whole calibration system was approximately 6.3%, and the uncertainty brought by the field surface measurement reached 5.4%, which might be the main reason for the relatively large deviation of band 13. This study verifies the feasibility of the vicarious calibration method at the Lake Qinghai RCS and provides the basis and reference for the subsequent on-orbit calibration of FY-3D/MERSI-II.

## 1. Introduction

Fengyun-3D (FY-3D) is a polar orbiting meteorological satellite launched on 15 November 2017. The main task of the Medium Resolution Spectral Imager II (MERSI-II) onboard FY-3D is to dynamically monitor the ocean, land, atmosphere and other environmental characteristics, especially the important atmospheric and environmental parameters such as clouds, aerosols, land surface characteristics and ocean surface characteristics [1].

Due to the aging of the instrument and external interference after satellite launch, the performance of remote sensors will experience an uncertain degradation. Taking Fengyun-3A (FY-3A) and Fengyun-3B (FY-3B) as examples, within two years after launch, the solar reflective bands decreased significantly. In particular, at band 8 (412 nm), the maximum attenuation reached 14% in the first year and 20% in the second year [2,3]. This result shows the importance of on-orbit calibration after satellite launch. Sun et al. proposed a new vicarious calibration method based on the surface bidirectional reflectance distribution function, the Second Simulation of a Satellite Signal in the Solar Spectrum (6SV) code and MODerate spectral resolution atmospheric TRANsmittance algorithm and computer model (MODTRAN4.0) for FY-3A/MERSI and FY-3B/MERSI [4]. Xu et al. solved the nonlinear response problem of the Fengyun-3C (FY-3C)/MERSI solar reflective bands and proposed an integrated method for on-orbit wide dynamic comprehensive radiometric calibration [5]. Wen et al. used a reflectance-based method to analyze the changes in the response of FY-3D/MERSI-II [6]. Wu et al. proposed an absolute radiometric calibration method based on FY-3D/MERSI-II lunar observation data. Meteorological satellites such as FY-3D are equipped with ocean color (OC) sensors [7]. However, no relevant research has conducted a separate surface vicarious radiometric calibration for the OC bands. To detect the response of the remote sensor at a low signal, we attempt to use the OC satellite calibration method to calibrate the OC bands of FY-3D/MERSI-II at Lake Qinghai.

The Coastal Zone Color Scanner (CZCS), launched on the Nimbus-7 satellite in 1978, was the first sensor used to measure OC data [8]. OC satellites are launched for the purpose of studying oceanic primary production on a global scale, which is unattainable via conventional in situ methods [9,10]. Currently, there are 6 sun-synchronous OC satellites in orbit, including the Terra/Moderate-resolution Imaging Spectroradiometer (MODIS), Aqua/MODIS, National Polar-Orbiting Partnership (NPP)/Visible Infrared Imaging Radiometer Suite (VIIRS), National Oceanic and Atmospheric Administration (NOAA)/VIIRS, Sentinel-3A/Ocean and Land Color Instrument (OLCI) and Sentinel-3B/OLCI [11]. Since the water surface radiance accounts for only approximately 10% of the total radiance received by the remote sensor, laboratory absolute calibration has difficulty achieving 0.5% accuracy for remote sensors [12]. The key to achieving the measurement accuracy is vicarious calibration [13].

Vicarious calibration is an on-orbit calibration method using a calibration field with known optical properties. Many scholars have proposed different vicarious calibration methods for OC sensors. Gordon first introduced the concept of vicarious calibration, which predicts the values of the spectral radiance measured by the sensor by combining the surface measurements and the atmospheric radiative transfer process [14]. Zhao et al. performed the absolute calibration of HaiYang-1B (HY-1B) data using Rayleigh scattering over clear ocean areas. The Rayleigh scattering calibration method has a total calibration error of 4.09% as determined by satellite sensors and mainly includes the uncertainty of ozone content, wind speed, air pressure, aerosol content and water-leaving radiance [15]. Wang et al. proposed near-infrared (NIR) and shortwave infrared (SWIR)-based on-orbit vicarious calibrations for NPP/VIIRS [16]. It is primarily assumed that the longest NIR band for ocean color processing is absolutely calibrated, and this approach it also utilized for Rayleigh scattering. Due to the limitations of field experiments, most vicarious calibration methods are independent of synchronous field-measured data. However, the calibration precision of the data based on field measurements is better than that based on other calibration methods. The field-measured data of the Lake Qinghai RCS were used to carry out vicarious calibration for the OC bands of FY-3D/MERSI-II to evaluate the decay condition of the satellite after launch and serve as a reference for other calibration methods.

This paper first introduces the principles and method of vicarious radiometric calibration. At the Lake Qinghai RCS, the water surface and atmospheric optical characteristics were obtained with high-precision instruments. Then the vicarious calibration coefficients were calculated with the radiative transfer model and satellite image data. The precision of the calibration method was evaluated by calibrating two representative satellite remote sensors, Aqua/MODIS and NPP/VIIRS, using the same calibration method. The results of the Dunhuang RCS calibration and lunar calibration were compared to the results of the Lake Qinghai RCS. The results verify the reliability and feasibility of the vicarious calibration for OC sensors and lay the foundation for further realization of domestic satellite calibration.

## 2. Calibration Method and Process

### 2.1. Method of Vicarious Radiometric Calibration

For OC sensors equipped with an onboard calibration system, the vicarious calibration is used as a supplementary test to check the function of the remote sensor, and for OC sensors, such as FY-3D/MERSI-II, vicarious calibration is the main calibration method [17].

For the ocean-atmosphere system, the top-of-atmosphere (TOA) radiance at the sensor can be expressed as [18,19],

(1) $$L_{t}(\lambda )=L_{r}(\lambda )+L_{a}(\lambda )+L_{ra}(\lambda )+t(\lambda )L_{wc}(\lambda )+T(\lambda )L_{g}(\lambda )+t(\lambda )L_{w}(\lambda )$$

where $L_{r}(\lambda )$ is the radiance of Rayleigh scattering in the absence of aerosols and can be exactly calculated by the observation geometry, pressure and wind speed [20]; $L_{a}(\lambda )$ is the radiance of aerosol scattering; and $L_{ra}(\lambda )$ is the radiance arising from multiple interactions of scattering by air molecules and aerosols [21]. The variables t(λ) and T(λ) represent diffuse transmittance of the atmosphere and atmospheric direct transmittance, respectively, which can be estimated using radiative transfer theory [22]. The quantities $L_{wc}(\lambda )$ and $L_{g}(\lambda )$ are the radiance resulting from whitecap and the radiance contributed from sun glitter, respectively [23]. The former can be computed by wind speed while the latter is generally avoided. $L_{w}(\lambda )$, derived from in situ data is the normalized water-leaving radiance [24].

**Figure 1:**
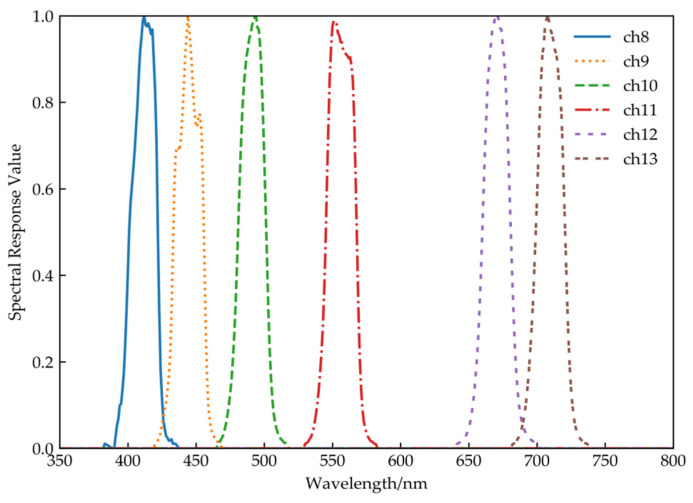
Spectral response function of the FY-3D/MERSI-II.

To obtain the average equivalent radiance $L_{i}(\lambda )$ of each channel, however, the obtained hyperspectral signal value still need to be convolved with the spectral response function of the sensor shown in Figure 1. The bands-averaged TOA radiance is given by,

(2) $$L_{t}(\lambda )=\frac{\int L_{total}(\lambda )S(\lambda )d\lambda }{\int S(\lambda )d\lambda }$$

where $S_{i}(\lambda )$ is the spectral response function.

As the average DN value of the pixel corresponding to the satellite image is extracted, the calibration coefficients g(λ) are computed, using,

(3) $$g(\lambda )=\frac{\pi L_{t}(\lambda )/F_{0}(\lambda )-Cal_{0}(\lambda )}{DN(\lambda )}g(\lambda )=\frac{\pi L_{t}(\lambda )/F_{0}(\lambda )-Cal_{0}(\lambda )}{DN(\lambda )}$$

where $F_{0}(\lambda )$ is solar irradiance on an average daily distance from the atmosphere,$Cal_{0}(\lambda )$ is the offset of the remote sensor value, and DN(λ) is the digital value of the remote sensors.

### 2.2. Process of Vicarious Radiometric Calibration

MODTRAN4.0 is an acronym for the MODerate spectral resolution atmospheric TRANsmittance algorithm and computer model, which was developed by the US Air Force Research Labs (AFRL) in collaboration with Spectral Sciences. This model can be used to calculate and simulate the spectral absorption, transmission, emission and scattering characteristics of the atmosphere.

**Figure 2:**
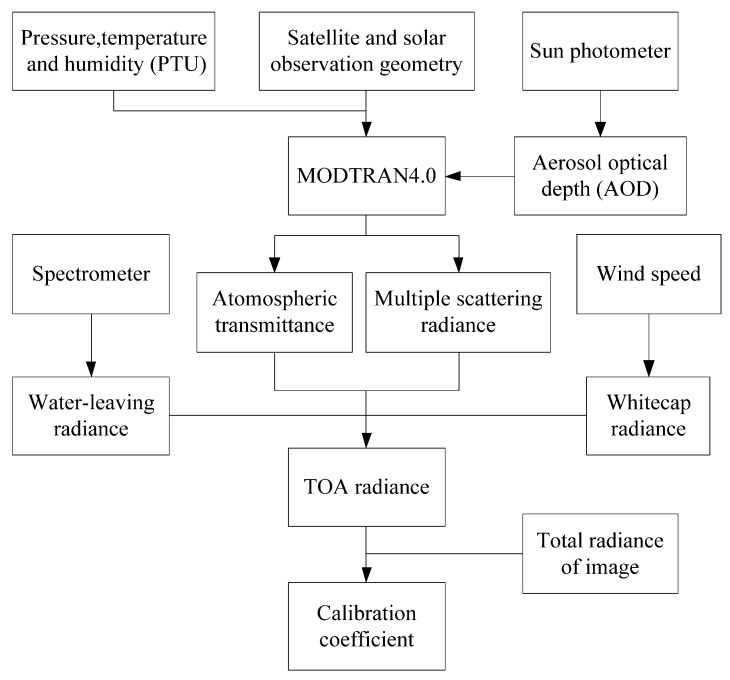
Flowchart of vicarious calibration method.

Figure 2 illustrates the process of vicarious radiometric calibration. The pressure, temperature and humidity, zenith and azimuth angles of the satellite and sun, aerosol optical depth (AOD) and water-leaving radiance data were collected and input into MODTRAN4.0, and the atmospheric transmittance and multiple-scattering radiance were output.

The detailed processing of field measurement data is shown as follows.

#### 2.2.1. Water-Leaving Radiance

The surface water-leaving radiance $L_{w}(\lambda )$ and remote sensing reflectance $R_{rs}$ are the main apparent optical properties (AOPs) of water [25]. Considering the operation of the field test and the characteristics of the water, the above water method was chosen. The above water method uses a rigorously calibrated spectrometer to measure the AOPs of the water surface with reasonable observation geometry and integral time. According to the principle of radiative transmission, electromagnetic waves will reflect, scatter and radiate spontaneously over the sea surface. Therefore, the sea surface radiance measured by the spectrometer is mainly derived from three radiance contributions during in situ measurement,

(4) $$L_{sea\;}(\lambda )=L_{w}(\lambda )+\rho \cdot L_{sky}(\lambda )+\Delta$$

where $L_{sea\;}(\lambda )$ is the total radiance of the sea surface; $L_{sky}(\lambda )$ is the radiance of the sky; ρ represents the reflectance of the water-air interface, which is related to the solar zenith angle, wind speed, wind direction and observation angle; and Δ represents external disturbances, including sun glitter, whitecap, and ship shadows.

**Figure 3:**
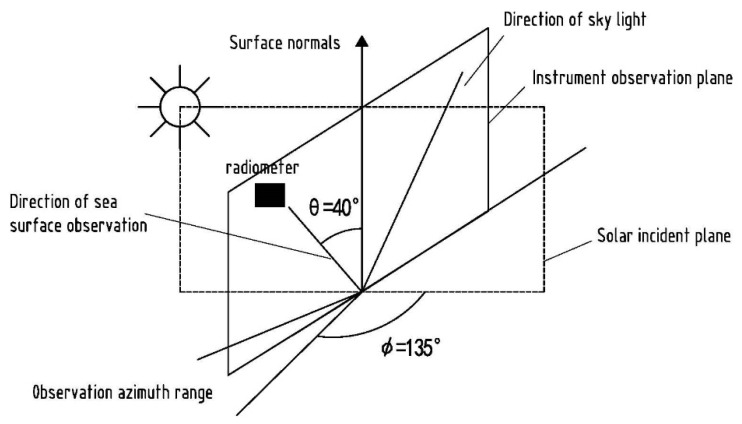
The geometric diagram of the above method.

In accordance with the observation angle recommended by the SeaWiFS marine optical specification shown in Figure 3 [26], the angle between the instrument observation plane and the solar incident plane must be between 90° and 135°, and the instrument observation direction should be at 40° to the normal direction of the sea surface [27,28]. The effects of sun glitter, whitecap and ship shadows are minimized only by measuring according to the above observation geometry and Equation (4) can be simplified as,

(5) $$L_{w}(\lambda )=L_{sea}(\lambda )-\rho \cdot L_{sky}(\lambda ).$$

Because the value of ρ is difficult to determine, according to the technical regulations of the marine optical survey, when the wind speed is less than 5 m/s and the solar zenith angle is between 30° and 60°, ρ can be determined to be 0.028.

To obtain the remote sensing reflectance, the white panel was used,

(6) $$R_{rs}(\lambda )=\frac{L_{w}(\lambda )}{E_{S}(\lambda )}=\frac{L_{sea}(\lambda )-\rho L_{sky}(\lambda )}{E_{S}(\lambda )}=\frac{L_{sea}(\lambda )-\rho \cdot L_{sky}(\lambda )}{\pi L_{p}(\lambda )}R_{p}(\lambda )$$

where $E_{S}(\lambda )$ is the surface incident irradiance, $L_{p}(\lambda )$ is the white panel radiance, and $R_{p}(\lambda )$ represents the white panel reflectance.

#### 2.2.2. Aerosol Optical Depth

Aerosol optical depth (AOD) is the basic parameter of radiative transmission and atmospheric correction, and affects the accuracy of radiometric calibration and atmospheric correction of satellite remote sensors [29]. The optical depth, single scattering albedo, phase function and refraction index of aerosols can be derived by the sun photometer [30].

According to the Beer-Bouguer-Lambert theorem [31], the monochromatic scattering of the direct irradiance of the sun that passes through the Earth’s atmosphere and reaches the ground can be expressed as,

(7) $$E(\lambda )=E_{0}(\lambda )d_{s}e^{-m[\tau_{r}(\lambda )+\tau_{a}(\lambda )]}T_{g}(\lambda )$$

where $E_{0}(\lambda )$ is the direct irradiance of the sun at the top of the Earth’s atmosphere at the average distance from the sun; $d_{s}$ is the correction factor of the distance between the sun and the Earth; *m* is the atmospheric mass; $\tau_{r}(\lambda )$ is the Rayleigh scattering optical depth; $\tau_{a}(\lambda )$ is the aerosol scattering optical depth; and $T_{g}(\lambda )$ is the transmittance of absorbed gas.

The components of gases with significant absorption are water vapor, carbon dioxide and ozone. Regarding the absorption of water vapor, there is a strong absorption zone at 936 nm, and the influence of water vapor on other bands is negligible. Regarding the absorption of ozone, the effect of each band should be considered. The effect on the absorption of carbon dioxide is also negligible at the bands of the solar photometer. According to the Beer theorem, ozone transmittance, $T_{oz}(\lambda )$, can be expressed as,

(8) $$T_{oz}(\lambda )=e^{-m\tau_{oz}(\lambda )}$$

where $\tau_{oz}(\lambda )$ is ozone optical depth.

Thus, Equation (7) can be reduced to,

(9) $$E(\lambda )=E_{0}(\lambda )d_{s}e^{-m[\tau_{r}(\lambda )+\tau_{a}(\lambda )+\tau_{oz}(\lambda )]}$$

Since the detection element of the solar spectrophotometer is a linear element, there is a linear relationship between dn(λ) output by the instrument and the solar irradiance [32]; therefore,

(10) $$\begin{array}{ll} \tau_{a}(\lambda ) & =\frac{1}{m}\ln\frac{E_{0}(\lambda )d_{s}}{E(\lambda )}-[\tau_{r}(\lambda )+\tau_{oz}(\lambda )]\\ & =\frac{1}{m}\ln\frac{dn_{0}(\lambda )d_{s}}{dn(\lambda )}-[\tau_{r}(\lambda )+\tau_{oz}(\lambda )] \end{array}$$

## 3. Field Campaign

Lake Qinghai (100°22′ E, 36°45′ N), the largest saline lake in China, is located on the northeastern Tibet Plateau [33]. As one of the most important RCSs in China, Lake Qinghai has a wide, open surface, considerable depth and a clean and pollution-free atmosphere, which are beneficial conditions for the test process. A field campaign was organized from 11 to 25 August 2018, at the Lake Qinghai RCS by the Anhui Institute of Optics and Fine Mechanics (AIOFM), Chinese Academy of Sciences (CAS), and the National Satellite Meteorological Center (NSMC).

**Figure 4:**
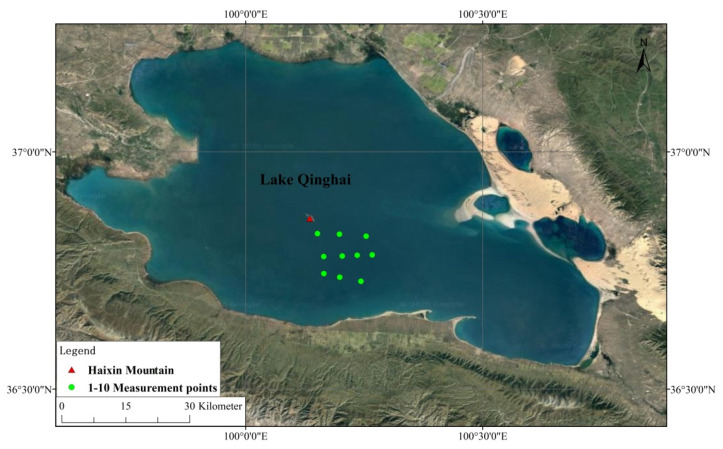
Synchronous observation points at Lake Qinghai.

As shown in Figure 4, an approximately 10 km × 10 km area southeast of Haixin Mountain in the middle of the lake was selected for the synchronous in situ observation experiment. By means of fixed-point stop-ship measurements, ten measurement points were selected to carry out field measurements in turn within an hour before, and after, the satellite overpass.

**Figure 5:**
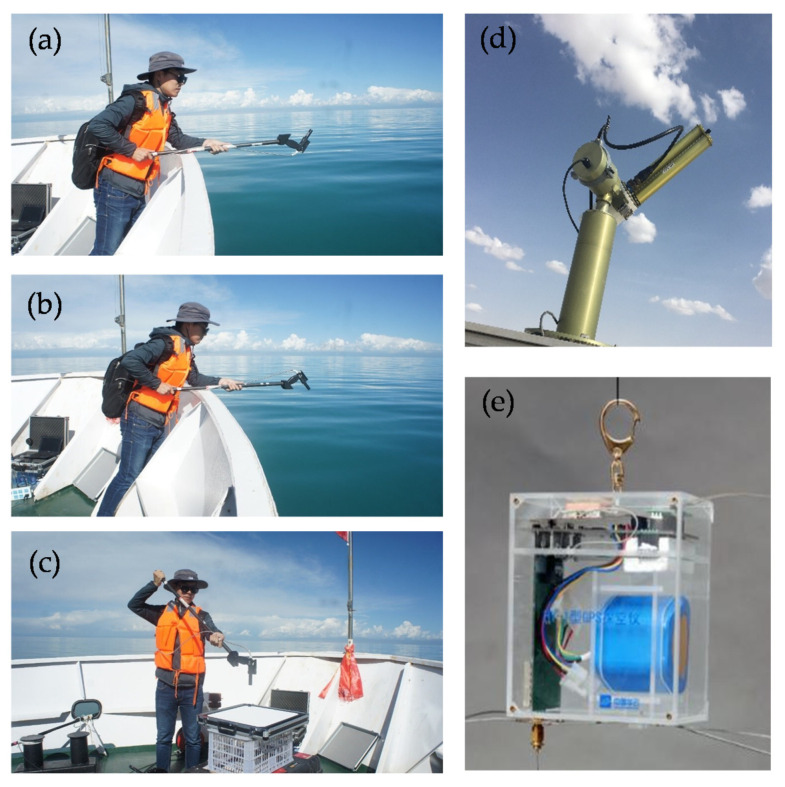
Observation equipment used in the experiment: (**a**–**c**) an ASD FieldSpec4 spectrometer for acquiring water surface parameters; (**d**) a CE-318 automated sun photometer for measuring atmospheric parameters; (**e**) a GPS radiosonde for obtaining PTU.

A variety of observation instruments, shown in Figure 5, were used to measure optical parameters such as water surface characteristics and atmospheric characteristics. Specifically, a field spectrometer (FieldSpec4) developed by Analytical Spectral Devices (ASD) is a portable instrument for measuring the visible (VIS) to NIR (300 nm to 2500 nm) spectrum. This instrument measures the surface and sky radiance to determine the water-leaving radiance, and the calibrated white panel radiance to determine the remote sensing reflectance.

An automated sun photometer called the CE-318/Sea-Viewing Wide Field-of-View Sensor Photometer Revision for Incident Surface Measurements (CE-318/SeaPRISM), developed by the CIMEL, automatically obtains the solar irradiance for the AOD. The sun photometer is a high-precision field solar and sky radiance measuring instrument that can be used to simultaneously measure the direct solar radiance and sky scattering radiance of different wavelengths. CE-318/SeaPRISM has nine observation bands with central wavelengths of 412 nm, 440 nm, 500 nm, 531 nm, 550 nm, 675 nm, 870 nm, 936 nm and 1020 nm and a bandwidth of 10 nm.

In addition, a GPS radiosonde, consisting of a global positioning system (GPS) module, a meteorological sensor, a radio transmitter and a battery, is also used to measure the pressure, temperature and humidity (PTU) information of the atmosphere. The GPS receiver module carried by the balloon was used to locate the balloon in real-time and calculate the wind direction and speed of the upper atmosphere. Atmospheric PTU sensors were used to measure meteorological elements of the atmosphere in real time. GPS positioning data and PTU data were sent to the ground receiving system by a radio transmitter. The ground receiving system received and processed the data to generate various required information.

## 4. Calibration Results

Table 1 shows the information for the solar and remote sensors at the moment of satellite transit, including date, time, zenith angle and azimuth angle. The relative azimuth of the sun and the FY-3D/MERSI-II was approximately 133° on August 14th and 19th. Satellite observations can be affected by sun glitter, which makes the results larger than the actual value. On the other hand, the relative azimuth angles on 18 August and 23 August were approximately 41°, and the effects of sun glitter were negligible. Thus, the data on 18 August and 23 August were considered valid and could be processed further.

### 4.1. In Situ Measurement Results

**Figure 6:**
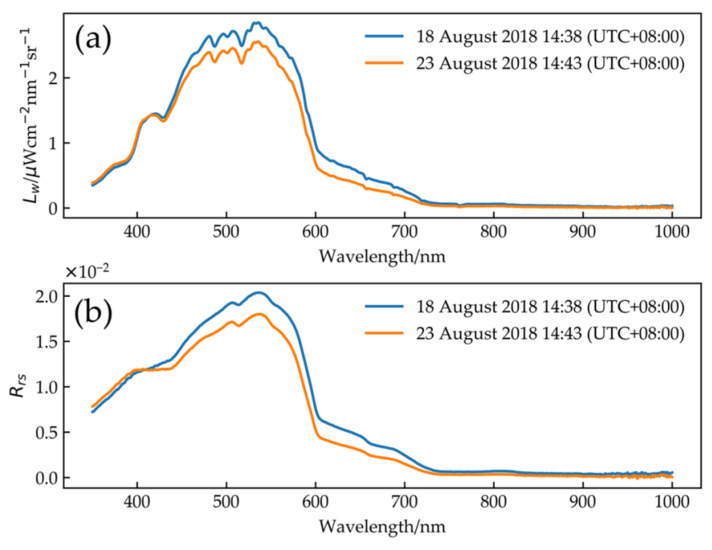
(**a**) Water-leaving radiance; (**b**) remote sensing reflectance.

Figure 6 shows the variation trend of water-leaving radiance and remote sensing reflectance. The maximum value of the two-day water-leaving radiance is taken near 545 nm, while the signal on the water after 700 nm is very weak.

**Figure 7:**
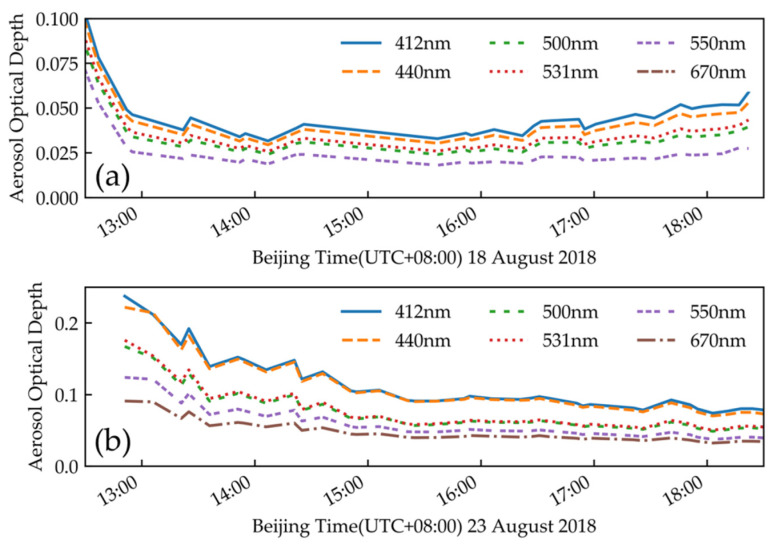
AOD (**a**) at 14:38 (UTC + 08:00), 18 August 2018; (**b**) at 14:43 (UTC + 08:00), 23 August 2018.

Figure 7 shows the AOD at 412, 440, 500, 531, 550 and 670 nm on 18 August and 23 August. The aerosol optical depth at the transit time of the satellite was small; thus, the atmosphere on these two days was relatively dry and clean, and suitable for vicarious calibration.

Whitecap radiance is the radiance produced by foam on the sea surface. According to the assumption that the reflection of the foam is similar to the Lambert reflection, the angle of the sun has little effect, and the whitecap radiance is almost ubiquitous in the satellite image. Gordon’s results show that whitecap radiance is independent of wavelength [34],

(11) $$L_{wc}(\lambda )=6.49\times 10^{-7}w_{s}{}^{3.52}F_{0}\cos(\theta_{0})/\pi$$

where $\theta_{0}$ is the solar zenith angle and $w_{s}$ is the wind speed.

**Figure 8:**
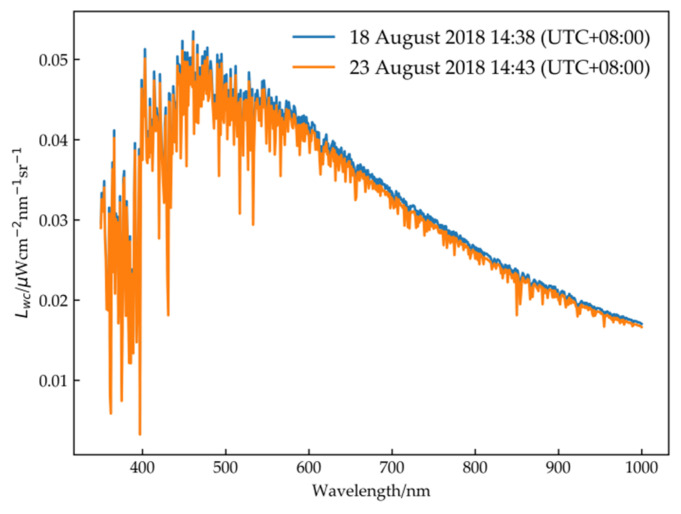
Whitecap radiance.

**Figure 9:**
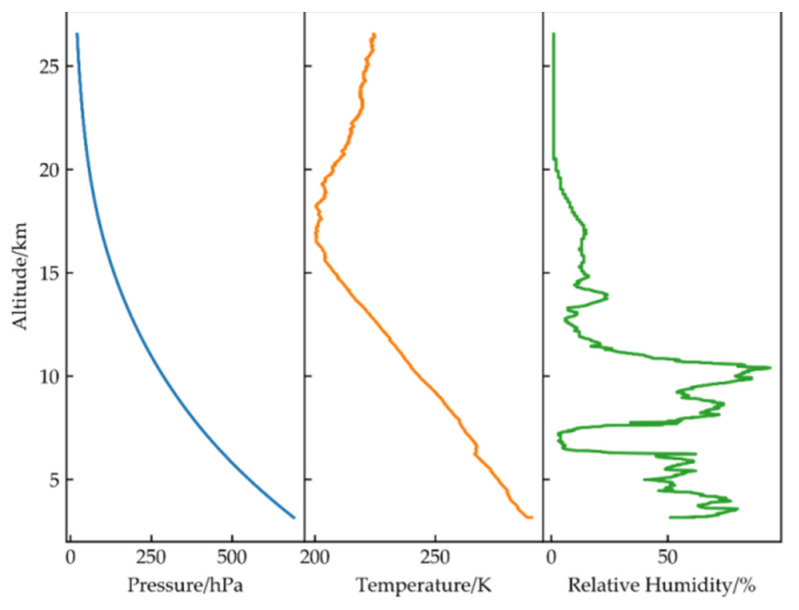
The curve of atmospheric PTU.

It can be seen from the formula that the higher the wind speed is, the greater the whitecap radiance. Thus, the experiment was carried out when the wind speed was less than 5 m/s. Figure 8 shows the whitecap radiance at various wavelengths at a wind speed of 4 m/s. The figure shows that the maximum value is only 0.05 $\mu W\cdot {\left(cm^{2}\cdot sr\cdot nm\right)}^{-1}$ at approximately 500 nm. Under calm sea conditions, the contribution of whitecap radiance has little influence on the vicarious calibration. Figure 9 shows the curve of each parameter changing with altitude. As seen from the PTU profiles, with increasing altitude, the pressure decreases, the temperature first decreases and then increases, while the relative humidity exhibits no obvious change pattern. We divided the PTU profiles into 32 layers to more accurately describe atmospheric characteristics at different altitudes.

### 4.2. Radiative Transfer Simulation

Table 2 lists the AOD value at 550 nm, water vapor content and ozone content at the moment of satellite transit. Combining the atmospheric and geometric parameters of the sun and the sensor in Table 1 allows the atmospheric transmittance and multiple scattering radiance to be simulated by the MODTRAN4.0 radiative transfer model.

**Figure 10:**
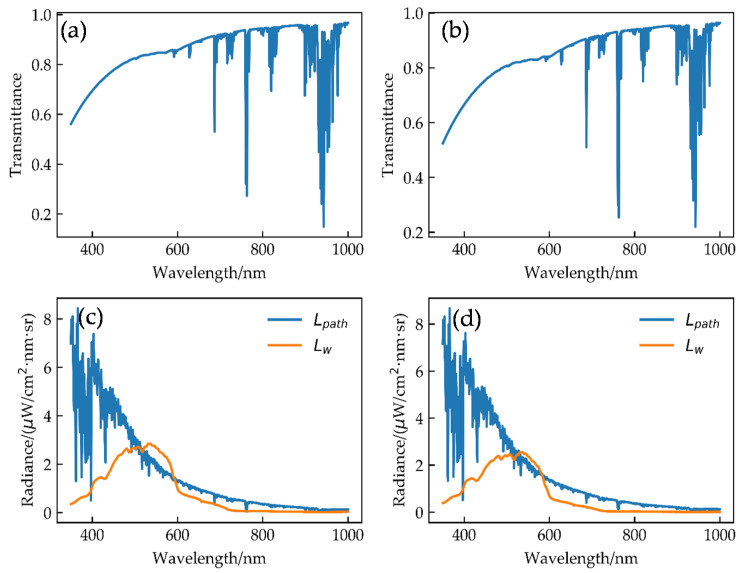
(**a**) Atmospheric transmittance at 14:38 (UTC + 08:00), 18 August 2018; (**b**) atmospheric transmittance at 14:43 (UTC + 08:00), 23 August 2018; (**c**) multiple scattering radiance and normalized water-leaving radiance at 14:38 (UTC + 08:00), 18 August 2018; (**d**) multiple scattering radiance and normalized water-leaving radiance at 14:43 (UTC + 08:00), 23 August 2018.

The results of MODTRAN4.0 are shown in Figure 10. With increasing wavelength, the atmospheric transmittance increases gradually, while the multiple scattering radiance decreases gradually. When the wavelength is greater than 700 nm, the normalized water-leaving radiance is almost zero. Therefore, the calibration range of OC bands at the sea surface is generally at the VIS bands.

### 4.3. FY-3D/MERSI-II Images

**Figure 11:**
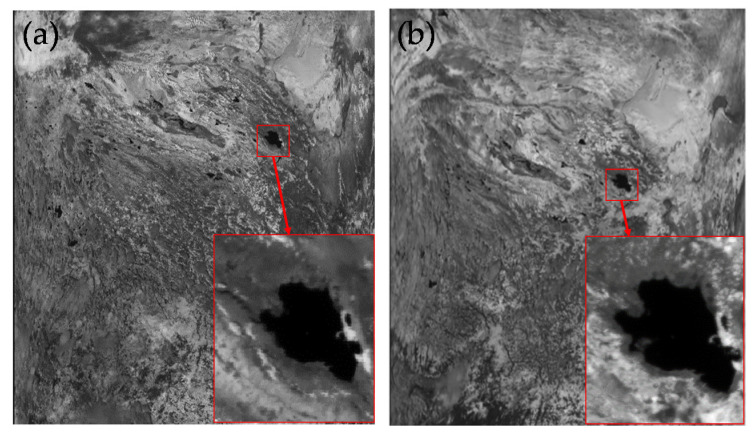
Satellite images: (**a**) 14:38 (UTC + 08:00), 18 August 2018; (**b**) 14:43 (UTC + 08:00), 23 August 2018.

Equation (3) shows that the calibration coefficients can be derived by extracting the satellite meter value. The image area of the satellite should be consistent with the synchronous observation area on the ground. Figure 11 shows satellite images taken on 18 and 23 August.

Lake Qinghai can be clearly seen in satellite images, indicating that there were no clouds covering the lake at the time. Taking the longitude and latitude of the measuring point as the center, the average value of the 5 × 5 pixel range in the satellite image was extracted as the satellite count value of each channel.

### 4.4. Calibration Coefficients of FY-3D/MERSI-II

After excluding the space observations value, the radiance calibration coefficients of each day can be obtained. Table 3 shows the calibration coefficients and the comparison with the prelaunch calibration coefficients.

The results for the calibration coefficients show an average difference of −17.056%, 2.278%, 4.799%, 0.061%, −0.353% and 11.963% when compared to the prelaunch calibration coefficients. In general, since the launch of the remote sensor, the calibration coefficients of most bands, except bands 8 and 13, are relatively stable, and the relative deviations are within 5%.

## 5. Comparison and Analysis

### 5.1. Accuracy Verification Based on Aqua/MODIS and NPP/VIIRS

As typical satellites in the world, Aqua/MODIS and NPP/VIIRS both have high calibration uncertainty. MODIS is an internationally recognized remote sensor for cross calibration that has high calibration accuracy, and its onboard calibration uncertainty is 2% [35]. The VIIRS calibration uncertainty is comparable to that of MODIS. Therefore, the synchronous observation data of Lake Qinghai were used to verify the accuracy of the vicarious calibration.

The geometric parameters and atmospheric parameters are listed in Table 4 and Table 5, respectively. According to the technical process shown in Figure 1, the total radiance of the satellite is simulated based on the atmospheric radiative transfer model.

The comparison results are shown in Table 6 and Table 7. The relative deviations of Aqua/MODIS for the first five OC bands are between 2.3% and 4.1%. In the red bands, the relative deviations are 11.9%, and 13.1%, respectively, which may be caused by the low water-leaving radiance signal from the two bands. The relative deviations of NPP/VIIRS for all OC bands range from 0.3% to 7.0%. The relative error at the red bands changes from−5% to 5%, indicating large fluctuations in these bands. However, the results show that the calibration method based on the Lake Qinghai RCS exhibits good calibration accuracy from 400 nm to 600 nm.

### 5.2. Comparison of Calibration Coefficients

On 18 and 23 August 2018, two effective calibration tests were completed at the Dunhuang RCS. The Dunhuang RCS is located in western Dunhuang city, Gansu Province, and has the advantages of flat terrain, uniform surface and good directionality. Internationally, it is agreed that the Dunhuang RCS is suitable for on-orbit radiation calibration of VIS and NIR remote sensors.

Detailed atmospheric and geometric information is listed in Table 8. The actual radiance received by the satellite was obtained by observing the surface reflectance, solar radiance and atmospheric radiance transmission characteristics of the satellite and combining them with the calculation of radiative transfer code 6SV. The calibration coefficients of the satellite are shown in Table 9.

**Figure 12:**
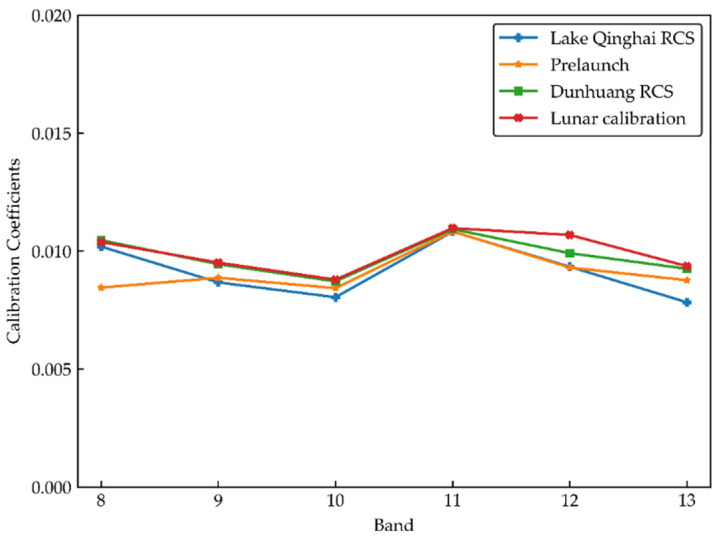
Calibration coefficients of three methods.

As lunar calibration is also a well-developed technique in reflective solar bands, Wu proposed an absolute radiometric calibration method based on FY-3D/MERSI-II lunar observation data [7]. The results from the lunar calibration compared with the prelaunch coefficient are listed in Figure 12 and Table 10.

Overall, the calibration results of the Lake Qinghai RCS are superior to those of the Dunhuang RCS and lunar calibration. Among them, the results of three on-orbit calibration methods show that there were significant errors at band 8, compared with the prelaunch laboratory calibration results, which were 17.1%, 19.2% and 22.9%. We suspect that the sensor decayed considerably at band 8 after launch. According to the pre-launch calibration by Xu et al. [36], the assessments conducted based on spherical integrating source (SIS) measurements indicate that band 8 exhibits a significant nonlinear behavior, which is consistent with the calibration results. The relative deviation of band 13 from the calibration result of Lake Qinghai is relatively large, reaching 12.0%. This difference may be due to the low signal of the water-leaving radiance at band 13, causing the measurement error to have a great impact on the calibration results. The uncertainty of each calibration process is assessed below. The relative deviations of other bands are within 5.0%, that of the Dunhuang RCS is within 6.2%, and that of lunar calibration is within 7.2%. A comparison with the existing research results verifies the consistency of the OC bands calibration results of FY-3D/MERSI-II and the feasibility of the calibration method.

### 5.3. Uncertainty Analysis

Many factors affect the calibration uncertainty of FY-3D/MERSI-II, including the measurement of the water-leaving radiance and the atmospheric parameters, the selection of aerosol model and the calculation error of the radiative transfer model.

Absolute radiometric calibration of ASD was carried out in the laboratory before the field test, in which the standard lamp of 1000 W spectral irradiance traced to the National Institute of Metrology and China (NIM) was used as the radiometric calibration light source. The calibration results showed that the uncertainty value of calibration at 400–800 nm was 2.0–2.1% [37]. The white panel used to measure the irradiance was calibrated by the NIM with an uncertainty value of 1.0%. Above-water method brought data of uncertainty in measurement and computation: 0.7–2.5% for water surface reflectance uncertainty [38], 1.9–2.9% for observation angle uncertainty [39], and 3.1% for environmental effect uncertainty [27].

AOD applies the same computation method as Aerosol Robotic Network (AERONET) and the nominal uncertainty value is 1.0–2.0% [40], including the uncertainty of the CE-318 calibration, extinction optical depth, Mie scattering, and Rayleigh scattering. The uncertainty value of the aerosol model is 1.9% [15]. For the OC wavelength, the uncertainty value 1.3% of absorbing gas is mainly from the ozone [41], while the uncertainty value of radiative transfer model is 1.0% [42]. The specific analysis of uncertainty is shown in Table 11.

According to the error transfer theory, the Quadratic sum of all the uncertainties above gives an uncertainty of 6.3% for the vicarious radiometric calibration. The computation equation is as follows,

(12) $$U_{total}=\sqrt{U_{L_{w}\;}^{2}+U_{AOD\;}^{2}+U_{Am\;}^{2}+U_{O_{3}}^{2}+U_{RTM\;}^{2}}$$

where $U_{L_{w}}$ is the uncertainty value of water-leaving measurement, $U_{AOD}$ is the uncertainty value of AOD, $U_{AM}$ is the uncertainty value of aerosol model, $U_{o_{3}}$ is the uncertainty value of ozone, and $U_{RTM}$ is the uncertainty value of radiative transfer model.

To reduce the uncertainty and improve the accuracy of the calibration, we will increase the frequency of calibration, improve the measurement accuracy and calibration accuracy of the instruments and establish an effective data processing model.

## 6. Conclusions

Due to the significant decay of the satellite after launch, it is an urgent and challenging task to calibrate the accuracy and stability of the radiation intensity of FY-3D, which was launched in November 2017. Vicarious radiometric calibration is the main method of calibration available. In this study, an attempt to calibrate of the OC bands for FY-3D/MERSI-II based on the Lake Qinghai RCS is proposed, which is different from the previous land calibration method at the Dunhuang RCS.

In this paper, we described the various instruments used in the field campaign, introduced the data processing in detail, and calculated the calibration coefficients via the radiative transfer model and satellite image extraction in reference to the calibration method described. The OC bands of FY-3D/MERSI-II are well calibrated at the Lake Qinghai RCS. The calibration coefficients of most bands are relatively stable and the relative deviations are within 5.0%. Aqua/MODIS and NPP/VIIRS were calibrated to verify the accuracy of the vicarious calibration using the same process as for FY-3D/MERSI-II radiometric calibration. The results show that the relative deviations of NPP/VIIRS are within 7.0%, and the calibration result of Aqua/MODIS is even within 4.1% from 400 nm to 600 nm. The calibration results were also compared with those of the Dunhuang RCS calibration and the lunar calibration. By comparing the results of the three on-orbit calibration methods and the existing research, we were able to determine that band 8 had a post-launch attenuation of 17.1%. Except for band 13, the relative deviations of the remaining bands were better than the existing calibration results. The relative deviation of band 13 reached 12.0%, which might be due to the measurement error of the water surface parameters. We evaluated the uncertainty of the whole calibration system and obtained a total uncertainty value of approximately 6.3%. The uncertainty value of field water surface measurement was 5.4%, which is the main source of calibration error.

This paper verified the feasibility of FY-3D/MERSI-II OC bands calibration at Lake Qinghai, and the results show that FY-3D/MERSI-II has good stability. The approach presented in this study, especially for remote sensing satellites without onboard calibration systems, can better monitor the variation in remote sensor response and ensure the quality of remote sensing data.

## Figures and Tables

**Table 1:** Geometric parameters during satellite overpass.

Date	Satellite	Beijing Time	Solar Zenith/(°)	Solar Azimuth/(°)	Satellite Zenith/(°)	Satellite Azimuth/(°)
14 August 2018	FY-3D	14:13	24.978	209.385	21.338	76.760
18 August 2018	FY-3D	14:38	28.989	219.850	18.627	261.370
19 August 2018	FY-3D	14:19	27.087	211.184	12.490	77.368
23 August 2018	FY-3D	14:43	31.288	221.120	27.653	262.060

**Table 2:** Atmospheric parameters at the time of overpass.

Date	Satellite	Beijing Time	AOD /(550 nm)	Water Vapor Column /(g·cm^−2^)	Ozone Column/(DU)
18 August 2018	FY-3D	14:38	0.035	1.5	276.5
23 August 2018	FY-3D	14:43	0.0863	1.09	279

**Table 3:** Calibration coefficients of FY-3D/MERSI-II at the Lake Qinghai RCS.

Bands	08-18	08-23	Mean	Prelaunch	Relative Deviation ^1^/(%)
8(412 nm)	0.010014	0.010366	0.01019	0.008452	−17.056
9(443 nm)	0.00864	0.008703	0.008672	0.008869	2.278
10(490 nm)	0.00813	0.007958	0.008044	0.00843	4.799
11(555 nm)	0.011383	0.010264	0.010824	0.01083	0.061
12(670 nm)	0.010055	0.008615	0.009335	0.009302	−0.353
13(709 nm)	0.008153	0.007504	0.007829	0.008765	11.963

^1^ Relative Deviation = (Prelaunch − Mean)/Mean × 100%.

**Table 4:** Geometric parameters of Aqua/MODIS and NPP/VIIRS.

Date	Satellite	Beijing Time	Solar Zenith/(°)	Solar Azimuth/(°)	Satellite Zenith/(°)	Satellite Azimuth/(°)
14 August 2018	NPP	14:28	26.763	218.04	19.99	218.71
18 August 2018	AQUA	14:53	31.2	226.33	39.88	263.005
19 August 2018	NPP	14:34	28.981	218.71	27.281	261.938

**Table 5:** Atmospheric parameters of Aqua/MODIS and NPP/VIIRS.

Date	Satellite	Beijing time	AOD /(550 nm)	Water Vapor Column /(g·cm^−^^2^)	Ozone Column/(DU)
14 August 2018	NPP	14:28	0.0876	1.97	275
18 August 2018	AQUA	14:53	0.035	1.5	276.5
19 August 2018	NPP	14:34	0.0804	1.54	278

**Table 6:** Comparisons of TOA radiance between simulated and Aqua/MODIS measured.

MODIS Bands	18 August 2018		Relative Deviation ^−1^ /(%)
	TOA Radiance $/\mu W\cdot {\left(cm^{2}\cdot \mathrm{sr}\cdot \mathrm{nm}\right)}^{-1}$		
	Sensor	Simulated	
8(412 nm)	7.161	6.995	−2.318
9(443 nm)	6.464	6.234	−3.558
10(488 nm)	5.546	5.32	−4.075
11(531 nm)	4.517	4.329	−4.162
12(551 nm)	4.188	4.071	−2.794
13(667 nm)	1.115	1.248	11.928
14(678 nm)	1.027	1.162	13.145

^1^ Relative Deviation = (Simulated − Sensor)/Sensor × 100%.

**Table 7:** Comparisons of TOA radiance between simulated and NPP/VIIRS measured.

VIIRS Bands	14 August 2018		Relative Deviation^1^ /(%)	19 August 2018		Relative Deviation^1^ /(%)
	TOA Radiance $/\mu W\cdot {\left(cm^{2}\cdot \mathrm{sr}\cdot \mathrm{nm}\right)}^{-1}$			TOA Radiance $/\mu W\cdot {\left(cm^{2}\cdot \mathrm{sr}\cdot \mathrm{nm}\right)}^{-1}$		
	Sensor	Simulated		Sensor	Simulated	
1(412 nm)	6.538	6.461	−1.178	6.621	6.493	−1.933
2(445 nm)	5.944	5.892	−0.875	5.812	5.793	−0.327
3(488 nm)	5.646	5.288	−6.341	5.310	5.026	−5.348
4(555 nm)	4.299	3.999	−6.978	3.889	3.685	−5.246
5(672 nm)	1.189	1.129	−5.046	1.042	1.102	5.758

^1^ Relative Deviation = (Simulated − Sensor)/Sensor ×100%.

**Table 8:** Atmospheric and geometric parameters of FY-3D/MERSI-II at the Dunhuang RCS.

Date	Beijing Time	AOD /(550 nm)	Water Vapor /(g·cm^−^^2^)	Ozone /(DU)	Solar Zenith /(°)	Solar Azimuth /(°)	Satellite Zenith /(°)	Satellite Azimuth /(°)
7 August 2018 12 August 2018	14:45	0.107	1.531	286	26.897	212.335	0.444	244.713
	14:50	0.103	1.543	286	28.859	213.883	9.509	260.443

**Table 9:** Vicarious calibration coefficient of FY-3D/MERSI-II at the Dunhuang RCS.

Bands	08-07	08-12	Mean	Prelaunch	Relative Deviation ^1^/(%)
8(412 nm)	0.01046	0.01046	0.01046	0.008452	−19.197
9(443 nm)	0.00947	0.00942	0.009445	0.008869	−6.098
10(490 nm)	0.00877	0.00866	0.008715	0.00843	−3.271
11(555 nm)	0.01103	0.01083	0.01093	0.01083	−0.915
12(670 nm)	0.01002	0.0098	0.00991	0.009302	−6.135
13(709 nm)	0.00936	0.00914	0.00925	0.008765	−5.243

^1^ Relative Deviation = (Prelaunch − Mean)/Mean × 100%.

**Table 10:** Relative deviation of three methods.

Bands	Relative Deviation/(%)		
	Lake Qinghai RCS	Dunhuang RCS	Lunar Calibration
8(412 nm)	−17.056	−19.197	−22.9
9(443 nm)	2.278	−6.098	−7.2
10(490 nm)	4.799	−3.271	−4.3
11(555 nm)	0.061	−0.915	−1.3
12(670 nm)	−0.353	−6.135	−14.8
13(709 nm)	11.963	−5.243	−6.8

**Table 11:** Uncertainty of vicarious calibration for FY-3D/MERSI-II.

Source	Uncertainty/(%)	Total Uncertainty/(%)
Water-leaving measurement		5.4
Calibration of ASD	2.1	
White panel	1.0	
Fresnel reflectance	2.5	
Observation angle	2.9	
Environmental effect	3.1	
AOD		2.0
Aerosol model		1.9
Ozone		1.3
Radiative transfer model		1.0
Quadratic sum		6.3

## Data Availability

Most data generated or analyzed during this study are included in the submitted article. Raw data and derived data supporting the findings of the study are also available from the corresponding author upon request.
